# Acetate metabolism in cancer cells

**DOI:** 10.1186/s40170-014-0027-y

**Published:** 2014-12-11

**Authors:** Aaron M Hosios, Matthew G Vander Heiden

**Affiliations:** Koch Institute for Integrative Cancer Research at Massachusetts Institute of Technology, Cambridge, MA 02139 USA; Dana-Farber Cancer Institute, Boston, MA 02115 USA

Macromolecule biosynthesis is required to duplicate cell components and support proliferation. Studies examining the nutrients used by cancer cells have focused on the contribution of glucose and glutamine carbon for biosynthesis, but the importance of other metabolic fuels is becoming apparent. Labeling of two-carbon units in newly synthesized lipids has been used to infer the nutrients that contribute to the acetyl-CoA pools in cells. Glucose- and glutamine-derived carbon are known to contribute extensively to *de novo* lipid biosynthesis, and in this issue Kamphorst *et al.* find that extracellular acetate can also contribute substantially to this process [1]. In normoxia, glucose and glutamine together account for the majority of lipogenic acetyl-CoA, but this fraction falls substantially in hypoxia [2,3]. In hypoxia, flux from glucose to citrate as a source of acetyl-coA decreases, and cells utilize alternative carbon sources to generate this metabolite. Glutamine can provide some of this carbon through reductive carboxylation of glutamine-derived α-ketoglutarate to citrate [2,4], but a substantial amount of acetyl-CoA is labeled by neither glucose nor glutamine tracing. Breakdown of lipids scavenged from the environment is one alternate source of acetyl-CoA, but mammalian serum also contains acetate, and the authors found that exogenous acetate could be incorporated into acetyl-CoA and become available for lipid biosynthesis. Surprisingly, for some cell lines in hypoxia, acetate may be a major contributor to acetyl-CoA.

Acetate is transported into cells by members of the monocarboxylate transporter family [5] where acetyl-CoA synthetases (ACSS) catalyze conversion to acetyl-CoA. Mammalian cells express mitochondrial and cytosolic forms of ACSS, and the authors cite emerging data from other groups that this enzyme can be important for growth of some tumors. Kamphorst *et al.* observe that acetate labels acetyl-CoA in hypoxic cells only, and it is interesting to consider whether this is due to increased acetate uptake or increased ACSS activity in low oxygen. Alternatively, it is possible that reduced synthesis of acetyl-CoA from glucose permits other sources of two-carbon units such as acetate to equilibrate with acetyl-CoA and have a higher fractional contribution to pools in cells.

For the labeling experiments acetate was provided to cells exogenously, but Kamphorst *et al.*’s findings raise the question of what sources of acetate exist *in vivo*. Acetate is present in human and murine blood at concentrations ranging from 50–200 μM [6-9] but has been estimated to be as high as 500–600 μM by some [10,11]. There can be substantial variability of acetate concentrations across species with higher levels observed in cows relative to human, perhaps explaining why Kamphorst *et al.* measure high levels of acetate in bovine serum [12]. Acetate in plasma is supplied by both exogenous and endogenous sources. Exogenous acetate delivered to tumors via the blood may derive from the diet, with a substantial portion generated by gut microbiome metabolism of intestinal contents [13]. Fasting induces the liver to generate ketones for metabolism by other tissues. Starvation itself reduces circulating acetate [14], presumably because dietary sources of acetate are lacking; however when ketones are consumed by cells they generate acetyl-CoA. Finally, exogenous acetate can be generated from the oxidation of ethanol. In some individuals, this can be a major source of acetate, and chronic drinkers may have circulating acetate concentrations as high as the millimolar range [10,15]. In the hours following ethanol consumption, blood acetate has also been observed to reach similar levels [16], but it has also been suggested that ethanol-derived acetate is primarily trapped in the liver by high ACSS activity [13].

Acetate may also be generated in a cell’s microenvironment. Protein deacetylases and acetyl-CoA hydrolase both generate acetate via hydrolysis reactions [14], suggesting that release of acetyl groups from endogenous sources may contribute to the acetate pool, and raising the possibility that some cells could produce acetate used by neighboring cells. Kamphorst *et al.* find that acetate’s contribution to lipid biosynthesis is greatest when cells are hypoxic, suggesting that substantial acetate utilization occurs when exogenous sources delivered by the blood are not available. This argues that acetate produced locally in the microenvironment would be the major source available in this context. Future work should clarify the use of circulating acetate by tumors, or whether the contribution of locally generated acetate predominates. It will also determine the importance of acetate use for proliferation and tumor progression, and elucidate why the use of acetate might be advantageous to cancer cells.
